# K18-hACE2 mice develop respiratory disease resembling severe COVID-19

**DOI:** 10.1371/journal.ppat.1009195

**Published:** 2021-01-19

**Authors:** Claude Kwe Yinda, Julia R. Port, Trenton Bushmaker, Irene Offei Owusu, Jyothi N. Purushotham, Victoria A. Avanzato, Robert J. Fischer, Jonathan E. Schulz, Myndi G. Holbrook, Madison J. Hebner, Rebecca Rosenke, Tina Thomas, Andrea Marzi, Sonja M. Best, Emmie de Wit, Carl Shaia, Neeltje van Doremalen, Vincent J. Munster

**Affiliations:** 1 Laboratory of Virology, National Institute of Allergy and Infectious Diseases, National Institutes of Health, Hamilton, Montana, United States of America; 2 Rocky Mountain Veterinary Branch, National Institute of Allergy and Infectious Diseases, National Institutes of Health, Hamilton, Montana, United States of America; The Peter Doherty Institute and Melbourne University, AUSTRALIA

## Abstract

SARS-CoV-2 emerged in late 2019 and resulted in the ongoing COVID-19 pandemic. Several animal models have been rapidly developed that recapitulate the asymptomatic to moderate disease spectrum. Now, there is a direct need for additional small animal models to study the pathogenesis of severe COVID-19 and for fast-tracked medical countermeasure development. Here, we show that transgenic mice expressing the human SARS-CoV-2 receptor (angiotensin-converting enzyme 2 [hACE2]) under a cytokeratin 18 promoter (K18) are susceptible to SARS-CoV-2 and that infection resulted in a dose-dependent lethal disease course. After inoculation with either 10^4^ TCID_50_ or 10^5^ TCID_50_, the SARS-CoV-2 infection resulted in rapid weight loss in both groups and uniform lethality in the 10^5^ TCID_50_ group. High levels of viral RNA shedding were observed from the upper and lower respiratory tract and intermittent shedding was observed from the intestinal tract. Inoculation with SARS-CoV-2 resulted in upper and lower respiratory tract infection with high infectious virus titers in nasal turbinates, trachea and lungs. The observed interstitial pneumonia and pulmonary pathology, with SARS-CoV-2 replication evident in pneumocytes, were similar to that reported in severe cases of COVID-19. SARS-CoV-2 infection resulted in macrophage and lymphocyte infiltration in the lungs and upregulation of Th1 and proinflammatory cytokines/chemokines. Extrapulmonary replication of SARS-CoV-2 was observed in the cerebral cortex and hippocampus of several animals at 7 DPI but not at 3 DPI. The rapid inflammatory response and observed pathology bears resemblance to COVID-19. Additionally, we demonstrate that a mild disease course can be simulated by low dose infection with 10^2^ TCID_50_ SARS-CoV-2, resulting in minimal clinical manifestation and near uniform survival. Taken together, these data support future application of this model to studies of pathogenesis and medical countermeasure development.

## Author summary

The disease manifestation of COVID-19 in humans ranges from asymptomatic to severe. While several mild to moderate disease models have been developed, there is still a need for animal models that recapitulate the severe and fatal progression observed in a subset of patients. Here, we show that humanized transgenic mice developed dose-dependent disease when inoculated with SARS-CoV-2, the etiological agent of COVID-19. The mice developed upper and lower respiratory tract infection, with virus replication also in the brain after day 3 post inoculation. The pathological and immunological diseases manifestation observed in these mice bears resemblance to human COVID-19. This suggests increased usefulness of this model for elucidating COVID-19 pathogenesis and for testing of countermeasures, both of which are urgently needed.

## Introduction

Severe acute respiratory syndrome coronavirus-2 (SARS-CoV-2) emerged in Hubai province in mainland China in December 2019, and is the etiological agent of coronavirus disease (COVID)-19 [1]. SARS-CoV-2 can cause asymptomatic-to-severe lower respiratory tract infections in humans, with early clinical signs including fever, cough and dyspnea [2, 3]. Progression to severe disease may be marked by acute respiratory distress syndrome (ARDS), with pulmonary edema, bilateral diffuse alveolar damage and hyaline membrane formation [4, 5]. Although primarily a respiratory tract infection, extra-respiratory replication of SARS-CoV-2 has been observed in kidney, heart, liver and brain in fatal cases [6–8]. Several experimental animal models for SARS-CoV-2 infection have been developed, including hamsters [9] ferrets [10] and non-human primates [11–14]. SARS-CoV-2 pathogenicity within these animal models ranges only from mild to moderate [9–14]. Additional small animal models that recapitulate more severe disease phenotypes and lethal outcome are urgently needed for the rapid pre-clinical development of medical countermeasures. Although the SARS-CoV-2 spike glycoprotein is able to utilize hamster angiotensin-converting enzyme 2 (ACE2) as the receptor of cell entry [9, 15], lack of species-specific reagents limit the usability of this model. As SARS-CoV-2 is unable to effectively utilize murine (m)ACE2 [16, 17], several models are currently under development to overcome this species barrier using a variety of strategies including transiently expressed human (h)ACE2, CRISPR/Cas9 modified mACE2, exogenous delivery of hACE2 with a replication-deficient viral vector and mouse-adapted SARS-CoV-2 [18–22].

K18-hACE2 transgenic mice were originally developed as a small animal model for lethal SARS-CoV infection. Expression of hACE2 is driven by a cytokeratin promoter in the airway epithelial cells as well as in epithelia of other internal organs, including the liver, kidney, gastrointestinal tract and brain. Infection with SARS-CoV led to severe interstitial pneumonia and death of the animals by day 7 post inoculation [19]. Here, we assess the susceptibility of K18-hACE2 transgenic mice as a model of severe COVID-19.

## Results

### Disease manifestation in SARS-CoV-2-inoculated K18-hACE2 mice

First, we determined the disease progression after SARS-CoV-2 inoculation. Two groups of 4–6 week-old K18-hACE2 transgenic male and female mice (15 each) were intranasally inoculated with 10^4^ (low dose group) and 10^5^ (high dose group) median tissue culture infectious dose (TCID_50_) SARS-CoV-2, respectively. In addition, one control group of two mice was intranasally inoculated with 10^5^ TCID_50_ γ-irradiated SARS-CoV-2.

**Fig 1:**
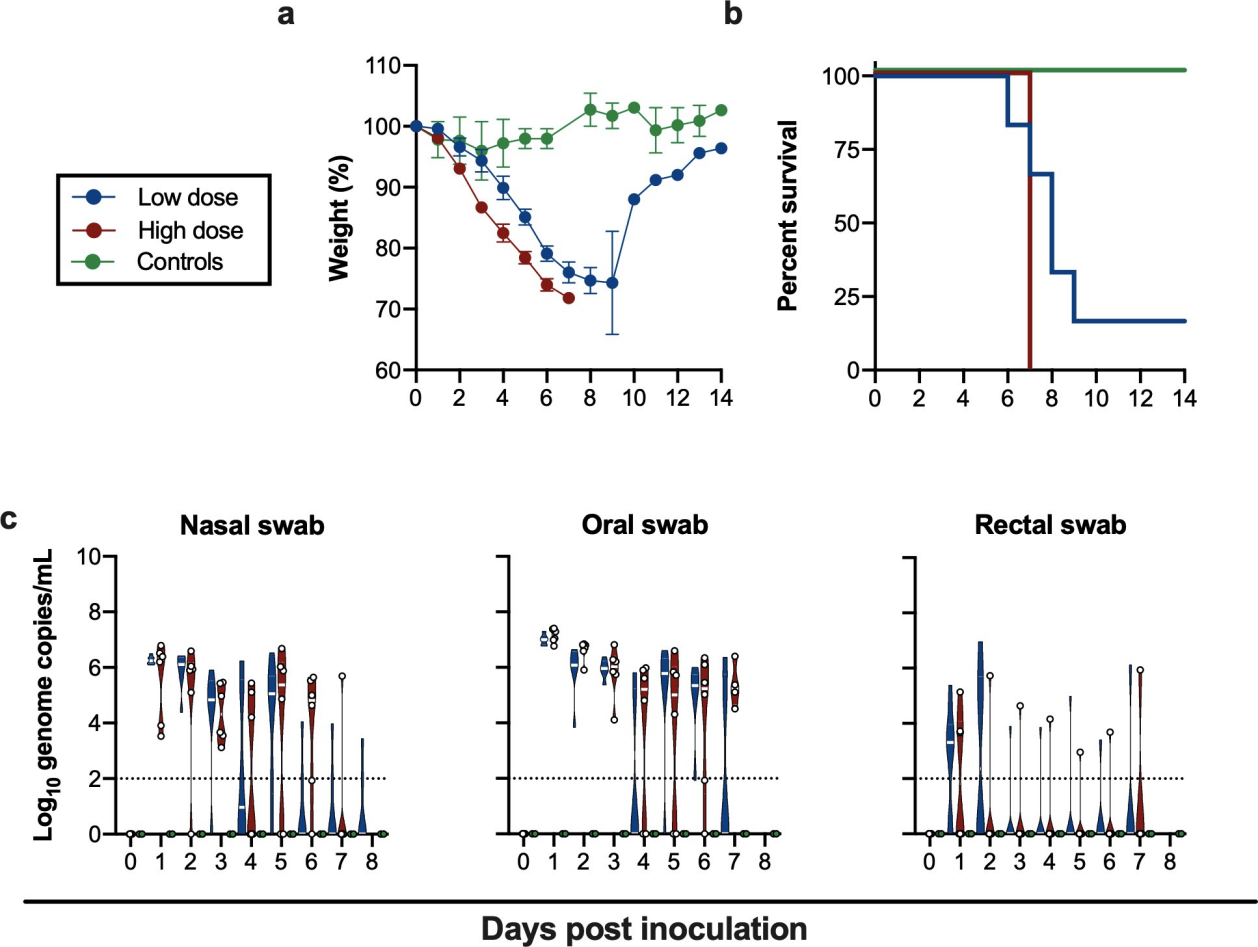
Inoculation of K18-hACE2 mice results in lethal infection and virus shedding. **. a**. Relative weight loss in mice after SARS-CoV-2 inoculation. The lines represent mean ± SEM. Wilcoxon matched-pairs rank test was conducted to compare weight differences between groups. **b**. Survival curves of mice inoculated with 10^4^ or 10^5^ TCID_50_ SARS-CoV-2_,_ or 10^5^ γ-irradiated SARS-CoV-2. **c**. Violin plot of viral load in nasal, oropharyngeal and rectal swabs with median as centre. Blue: 10^4^ TCID_50_ (low dose animals, n = 6); red: 10^5^ TCID_50_ (high dose animals, n = 6); green: 10^5^ TCID_50_ γ-irradiated (control animals, n = 2); dotted line = limit of detection.

Irrespective of SARS-CoV-2 inoculation dose, mice uniformly started losing weight at 2 days post inoculation (DPI) (Fig 1A), with a significantly higher weight loss observed in the high dose group, suggesting a dose-response relationship, (p = 0.02, Wilcoxon matched-pairs rank test). No difference in weight loss between male and female animals within the same dose group was detected (S1B Fig). In addition to weight loss, lethargy, ruffled fur, hunched posture and labored breathing were observed throughout the course of infection in each animal. Mice were monitored for signs of neurological disease (circling, rolling, hyperexcitability, convulsions, tremors, weakness, or flaccid paralysis of hind legs), and no neurological symptoms were observed in any of the animals. Within the high dose group all animals reached euthanasia criteria by 7 DPI, however, in the low dose group five out of six animals reached euthanasia criteria 5–9 DPI and one animal recovered (Fig 1B). The animal size used in this study was too small to draw major conclusions on survival. The control animals inoculated with γ-irradiated SARS-CoV-2 did not lose weight and remained free of disease symptoms.

### Viral shedding in SARS-CoV-2-inoculated K18-hACE mice

To gain an understanding of dose-dependent virus shedding patterns of SARS-CoV-2 in infected K18-hACE2 mice, daily nasal, oropharyngeal and rectal swabs were obtained until 11 DPI. Viral RNA was detected in all three (Fig 1C). SARS-CoV-2 shedding from the respiratory tract was observed in all inoculated animals. Viral load in oropharyngeal and nasal swabs reached up to ~10^6^ and ~10^7^ copies/mL, respectively, and viral RNA could be detected up to 7 and 8 DPI. Rectal shedding was observed in both inoculated groups, but not in all animals, and was lower compared to respiratory shedding. Importantly, no viral RNA could be detected in swabs obtained from control mice inoculated with γ-irradiated SARS-CoV-2, suggesting viral RNA detected as early as 1 DPI was directly associated with active virus replication and did not originate from inoculum (Fig 1C). No sex-dependent differences in shedding pattern were seen (S1C Fig).

### Tissue tropism of SARS-CoV-2-inoculated K18-hACE mice

**Fig 2:**
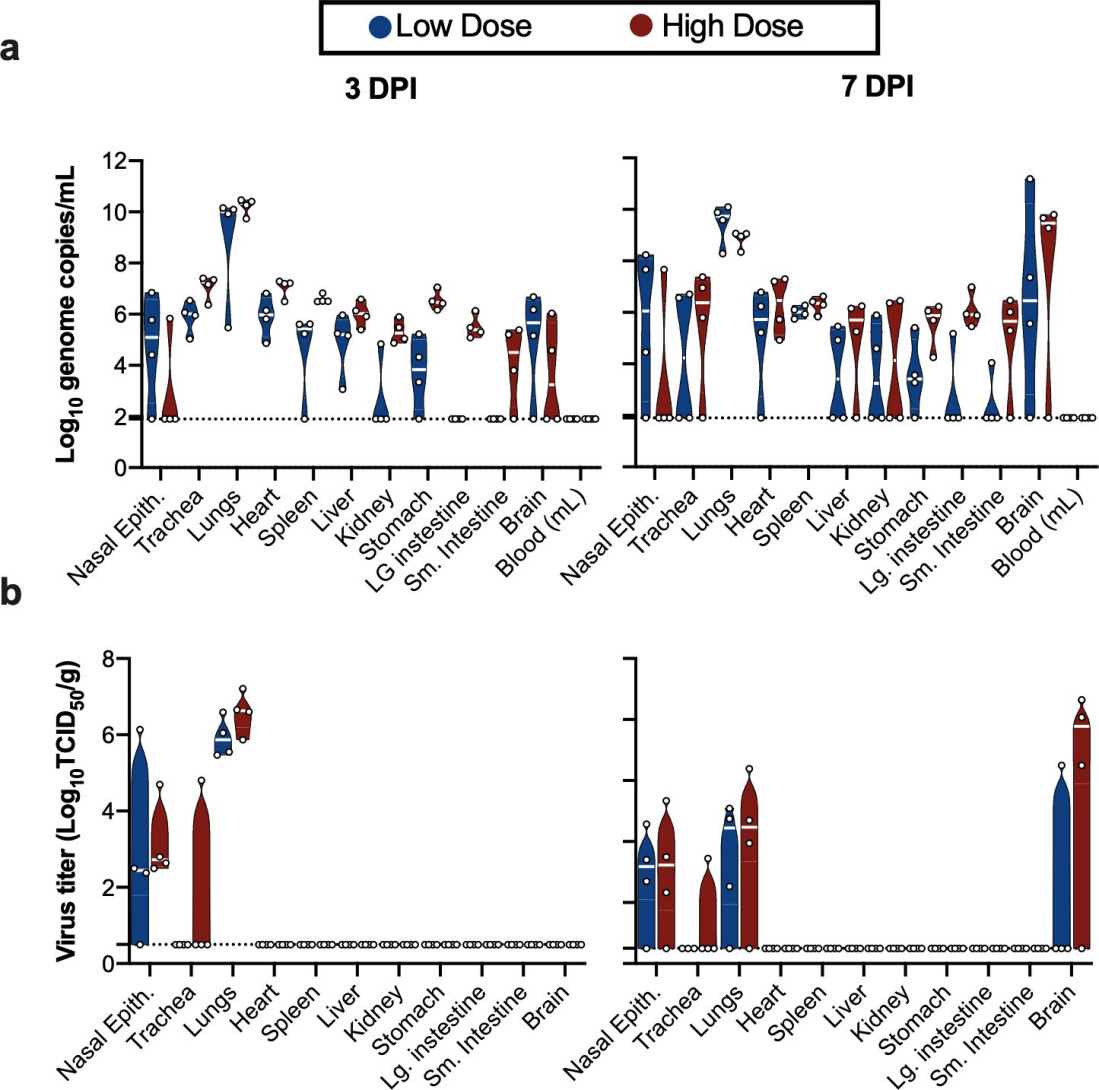
SARS-CoV-2 tissue tropism in K18-hACE mice. **a.** Violin plot of viral load in tissues quantified by RT-qPCR with median as centre. **b.** Violin plot of infectious SARS-CoV-2 titers in tissues, with median as centre. Blue: 10^4^ TCID_50_ (low dose animals, n = 6); red: 10^5^ TCID_50_ (high dose animals, n = 6); green: 10^5^ TCID_50_ γ-irradiated (control animals, n = 2); dotted line = limit of detection.

We next assessed tissue tropism and viral replication of SARS-CoV-2 in K18-hACE2 mice (Fig 2A). Viral genomic RNA was detected in almost all tissues; however, no viremia was observed. At 3 and 7 DPI, the highest viral load was found in lung tissue (~10^10^ genome copies/g). Viral RNA in brain tissue was increased at 7 DPI compared to 3 DPI (from ~10^5^ to 10^10^ genome copies/g) (Fig 2A). When assessing infectious virus, at 3 DPI, it was only detected in respiratory tract tissues, with high infectious titers observed in nasal epithelium and lungs in both the low dose and high dose groups. At 7 DPI, infectious virus was detected in respiratory tract as well as brain tissue (Fig 2B). Together, these data suggest that either SARS-CoV-2 initially exclusively targets the respiratory tract with a secondary central nervous system (CNS) involvement after 3 DPI, or, due to the route of challenge employed here, the infection starts in the respiratory tract and only reaches the brain after 3 DPI.

### Histological changes and viral antigen distribution in SARS-CoV-2-inoculated K18-hACE mice

**Fig 3:**
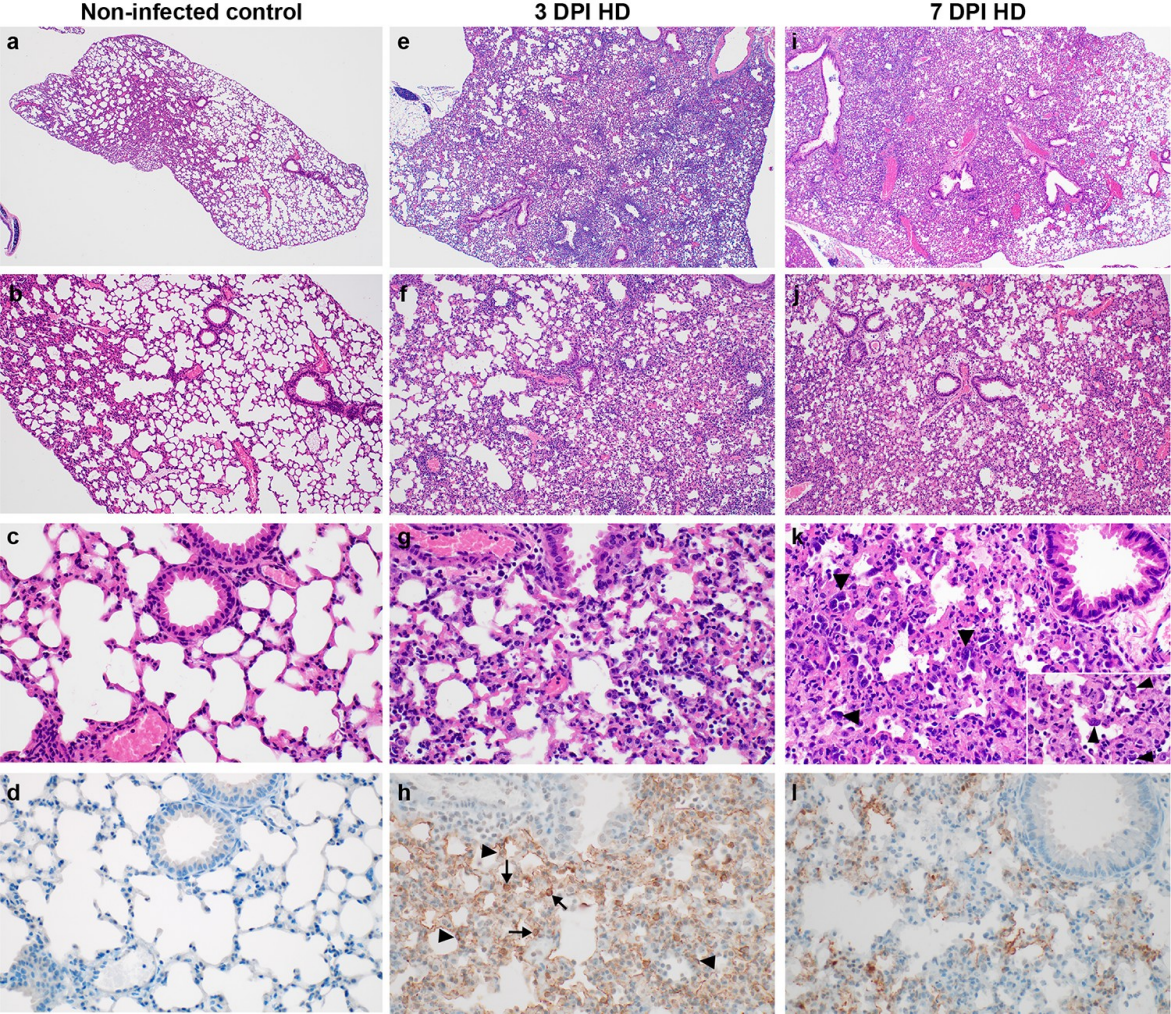
Pathological changes in lungs of K18-hACE mice inoculated with SARS-CoV-2 at 3 and 7 DPI. a, b, c, d. γ-irradiated SARS-CoV-2 inoculated control lungs appear normal and lack SARS-CoV-2 antigen immunoreactivity. **e, f, g**. Inflammation at 3 DPI, characterized by perivascular and septal infiltration by neutrophils, macrophages, lymphocytes, and edema. **i, j, k.** Multifocal interstitial pneumonia at 7 DPI, characterized by type II pneumocyte hyperplasia (arrowheads), alveolar and perivascular inflammation, fibrin, edema, syncytial cells (inset arrowheads) and single cell necrosis. **h, l.** SARS-CoV-2 antigen immunoreactivity in type I (arrowheads) and type II pneumocytes (arrows) at 3 and 7 DPI. HD: high dose (10^5^ TCID_50_ SARS-CoV-2). Magnification: a, e = 40 x; b, f = 100 x; c, g, h = 400 x, inset 1000 x.

At 3 and 7 DPI, four animals from each group were euthanized and necropsies performed. On both days, gross lung lesions were observed in all mice from each group with up to 80% of the lung tissue affected. Histologically, all mice developed pulmonary pathology following inoculation with SARS-CoV-2. As compared to controls (Fig 3A–3D) lungs developed inflammation by 3 DPI characterized by perivascular lymphocytes and alveolar septa thickened by neutrophils, macrophages and edema (Fig 3E–3G). At 7 DPI, mice exhibit multifocal, and often peripheral, pulmonary pathology consistent with interstitial pneumonia characterized by type II pneumocyte hyperplasia, septal, alveolar and perivascular inflammation comprised of lymphocytes, macrophages and neutrophils, variable amounts of alveolar fibrin and edema, frequent syncytial cells and single cell necrosis (Fig 3I–3K). Terminal bronchioles were similarly affected and, in the most severely affected areas, fibrin and necrotic debris occluded the lumen (data not included). Immunohistochemistry (IHC) demonstrated SARS-CoV-2 viral antigen in both type I and type II pneumocytes from 3 and 7 DPI mice (Fig 3H and 3l).

**Fig 4:**
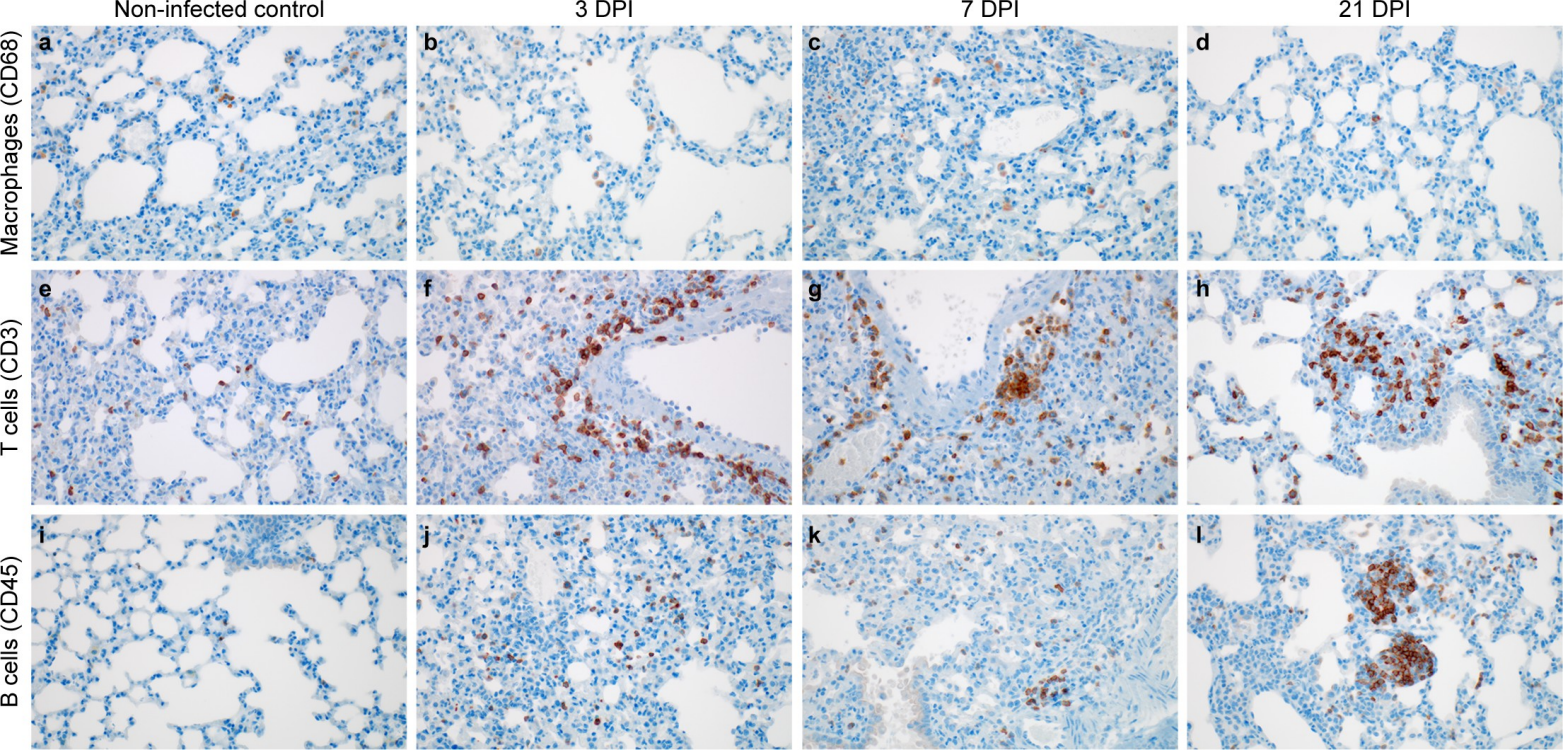
Infiltration of innate and adaptive immune-cell populations in the lungs of SARS-CoV-2 infected mice. a, e, i, non-infected control. b, f, j,105 TCID50 3 DPI. c, g, k, 105 TCID50 7 PDI d, h, l, survivor animal 21 DPI. a. γ-irradiated SARS-CoV-2 inoculated controls with few macrophages (brown). b, c. Increased macrophages at 3 and 7 DPI. d. Macrophages present at end of study. e. Scattered T cells (brown) in the γ-irradiated SARS-CoV-2 inoculated controls. f, g. T cells are increased in perivascular tissue and alveolar septa at 3 and 7 DPI. h. T cells forming lymphoid aggregates with B cells in perivascular tissues. i. B-cells (brown) are few in the γ-irradiated SARS-CoV-2 inoculated controls. j,k. B cells are increased in alveolar septa at 3 and 7 DPI. l. B cells forming lymphoid aggregates with T cells in perivascular tissues. Magnification: a-l = 400 x.

We evaluated the localized infiltration of innate and adaptive immune cell populations at 3 and 7 DPI, as compared to control mice and the survivor at 21 DPI. Immunoreactive macrophages (CD68+) were present in the γ-irradiated SARS-CoV-2 inoculated controls at 3 DPI; however, there was a slight increase in the presence of alveolar macrophages at 7 DPI (Fig 4A–4C). At 21 DPI alveolar macrophages were individually scattered throughout the lung (Fig 4D) as well as clustered in persistent foci of inflammation (S2C and S2E Fig). We next assessed lymphocyte infiltration into the lung in more detail. T cells appeared evenly distributed throughout the γ-irradiated SARS-CoV-2 inoculated controls (Fig 4E). At 3 and 7 DPI T cells formed perivascular cuffs and were increased in alveolar septa (Fig 4F and 4G). B cell numbers were low in the controls (Fig 4I), increased in alveolar septa at 3 DPI (Fig 4J), and started to cluster by 7 DPI (Fig 4K). At 21 DPI, T cells were present throughout the lung section and formed lymphoid aggregates with B cells in perivascular tissues (Fig 4H–4L).

**Fig 5:**
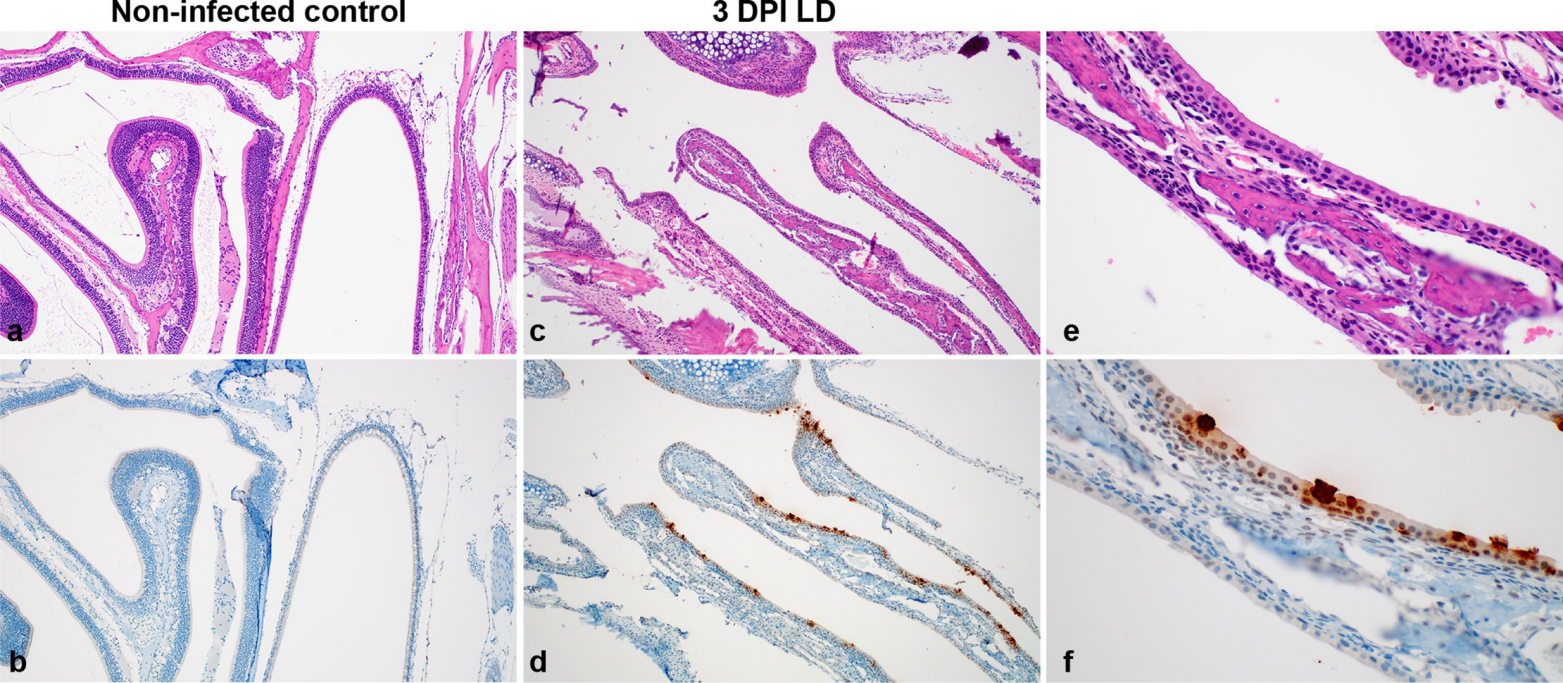
Histologic features of nasal turbinates of SARS-CoV-2 infected mice. **a**. Normal nasal turbinates from γ-irradiated SARS-CoV-2 inoculated control mouse lined by olfactory and respiratory epithelium. **b**. Absence of SARS-CoV-2 antigen immunoreactivity in respiratory and olfactory epithelial cells of low dose inoculated animal at 3 DPI. **c.** 3 DPI nasal turbinates lack inflammation but **d.** exhibit cytoplasmic immunoreactivity (brown) of respiratory epithelial cells for SARS-CoV-2 antigen. **e**. Higher magnification of respiratory epithelium. **f**. Higher magnification showing SARS-CoV-2 antigen immunoreactivity in ciliated respiratory epithelial cells. Magnification: a, b, c, d = 100 x; e, f = 400 x.

Histologic lesions were not observed in the nasal turbinates at 3 and 7 DPI (Fig 5A, 5C and 5E); however, IHC staining revealed multifocal SARS-CoV-2 antigen immunoreactivity in ciliated and non-ciliated respiratory epithelial cells at 3 DPI (Fig 5D–5F).

**Fig 6:**
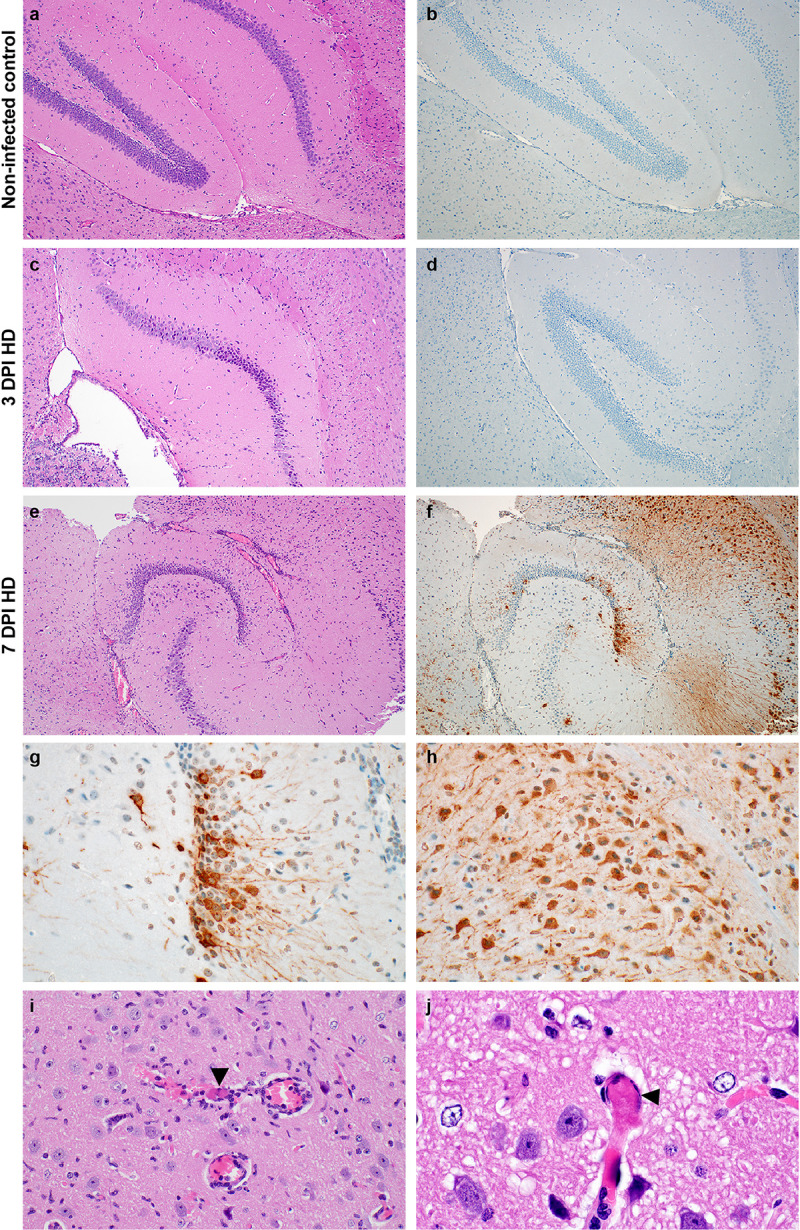
Neurotropism of SARS-CoV-2 in infected mice at 7 DPI. **a-b** Normal hippocampus in a γ-irradiated SARS-CoV-2 inoculated control mouse. **c**. No inflammation is noted and **d**. there is no detection of SARS-CoV-2 antigen at 3 DPI. **e.** Generalized increase in cellularity of the cerebral cortex and hippocampus and the meninges are mildly expanded by edema and inflammatory cells at 7 DPI. **f**. SARS-CoV-2 antigen immunoreactivity (brown) throughout the cerebral cortex and hippocampus. **g and h**. SARS-CoV-2 antigen immunoreactivity in neurons of the hippocampus and cerebral cortex. **i**. A small caliber vessel in the cerebral cortex contains a microthrombus (arrowhead) surrounded by hemorrhage and inflammatory cells which infiltrate the adjacent neuropil; there are increased glial cells throughout the image. **j**. Another microthrombus (arrowhead) in a small caliber vessel. Magnification: a, b, c, d, e, f = 100 x; g, h, i = 400 x; and j = 1000 x.

Sections of brain were histologically normal in the γ-irradiated SARS-CoV-2 inoculated controls and in all low dose and high dose mice at 3 DPI (Fig 6A–6D); however, at 7 DPI, in low and high dose animals lesions ranged from minimal to moderate and included lymphocytic perivascular cuffing, gliosis, meningeal and perivascular inflammation, edema and rare cerebral microthrombi (Fig 6E, 6I and 6J). Abundant SARS-CoV-2 antigen was detected in neurons of the cerebral cortex and hippocampus (Fig 6F–6H).

Dose-dependent differences in histological changes in the lungs and brains of these mice was limited. There was a minor increase in perivascular inflammation within the lungs of the high dose inoculated group over the low dose group at 3 DPI. At 7 DPI, 2 out of 7 of the low dose mice developed inflammation or necrosis of the bronchioles, whereas, all 8 high dose mice were affected. There were no differences in the brain between the low and high dose inoculated mice at 3 DPI. At 7 DPI, 3 out of 7 low dose mice exhibited minimal perivascular cuffing, while 7 out of 8 of the high dose mice had minimal to mild perivascular cuffing with inflammatory cells extending into the adjacent neuropil, gliosis, and, in two cases, microthrombi within capillaries (S1 Table).

### Rapid humoral immune response in SARS-CoV-2-inoculated K18-hACE mice

**Fig 7:**
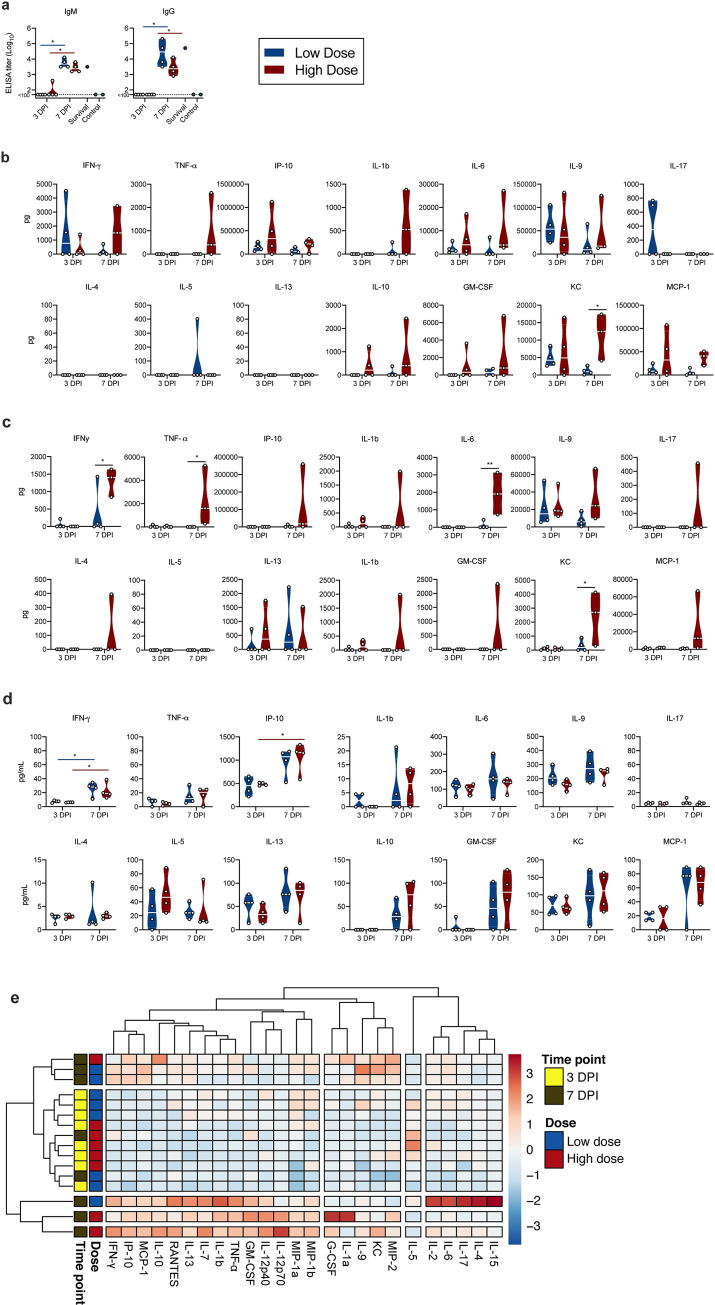
Humoral and cytokine/chemokine responses to SARS-CoV-2 infection in K18-hACE mice. **a**. IgM and IgG antibody titres against SARS-CoV-2 spike ectodomain by ELISA in serum. White line represents geometric mean of end point dilutions per study group. Dotted line represents limit of detection. **b, c, d**. Four-fold serial-diluted brain homogenate, lung homogenate and serum of selected cytokines/chemokines in K18-hACE mice challenged with SARS-CoV-2 measured on Bio-Plex 200 instrument (Bio-Rad) using Milliplex Mouse Cytokine/Chemokine MAGNETIC BEAD Premixed 25 Plex Kit (Millipore). Violin plots are depicted with individual values and median of all mice. Two-tailed Mann-Whitney’s rank test was used to compare differences between groups. e. Heatmap showing cytokine titers clusters based on DPI and dose of inoculation.

We next investigated two key aspects of the anti-viral immune response. To assess B-cell response and class-switching, the presence of SARS-CoV-2 spike-specific immunoglobulin (Ig)G and IgM antibodies in serum obtained at 3 and 7 DPI was investigated using ELISA. By 3 DPI, one mouse in the high dose group was positive for IgM and no mice were positive for IgG. In contrast, both spike-specific IgM and IgG were found in sera of all mice at 7 DPI (Fig 7A). IgM and IgG titers of one surviving animal at 21 DPI were comparable to those at 7 DPI.

### Rapid systemic upregulation of proinflammatory cytokines and chemokines in SARS-CoV-2-inoculated K18-hACE mice

To investigate the immune response further we utilized multiplex cytokine analysis to characterize the inflammatory status and identify key patterns. Cytokine levels were analyzed in lung and brain tissues collected at 3 DPI and 7 DPI for both low and high dose groups (Fig 7B and 7C). Pro-inflammatory cytokines including T helper (Th)1-mediated cytokines interferon (INF)-γ and tumour necrosis factor (TNF)-α, as well as chemoattractant granulocyte-macrophage colony-stimulating factor (GM-CSF) and monocyte chemoattractant protein-1 (MCP-1) showed increased levels in the high dose 7 DPI animals. In brain samples, the elevated levels of INF-γ and TNF-α were significantly higher in high dose animals, as compared to low dose. Additionally, on 7 DPI a significantly higher level of KC (C-X-C motif chemokine ligand (CXCL)-1) was observed in the high dose animals as compared to those infected by the lower dose in both brain and lung samples. Levels of interleukin (IL)-6 were elevated in the lungs of high dose animals compared to low dose animals, at both time points. However, this effect was not significant. In the brain, no IL-6 was observed for low dose animals; however, significant change in levels were seen for high dose animals at 7 DPI. No significant correlation between cytokine levels and viral RNA measured in the lung tissue was seen (S3A Fig). In the brain, the strongest positive correlation with viral RNA levels was observed for the expression of IL-6 (S3B Fig).

Interestingly, while serum cytokine levels at 3 DPI showed only slight changes, strong upregulation was observed for multiple cytokines and chemokines by 7 DPI (Fig 7D and S3C Fig). A strong increase in IFN-γ (both doses, p = 0.0268, 0.0268) and TNF-α, (though not statistically significant) was observed. In addition, there was also an upregulation of proinflammatory and chemoattractant cytokine IFN-γ-induced protein (IP)-10 CXCL10 (high dose, p = 0.0268). Interestingly, no trend of upregulation of Th2 anti-inflammatory cytokines IL-4 and IL-5 was seen, but increased levels of IL-10 were observed at 7 DPI in both groups, which has been shown to have an anti-inflammatory regulatory function in mediating antiviral responses [23]. In addition, GM-CSF, KC and monocyte chemoattractant protein-1 (MCP-1 (C-C motif chemokine ligand (CCL1)) were detected systemically and at increased levels at 7 DPI, further indicating a systemic recruitment of inflammatory and innate immune cells to sites of infection. Of note, this model did not recapitulate the increase of systemic IL-6 observed in severe COVID-19 patients [24] in either dose or timepoint. When comparing the overall systemic cytokine profile of each animal, it became obvious that the observed cytokine upregulation was more closely linked with time post inoculation than with viral dose. We observed 3 clusters, which showed a clear time-correlation and did not detect significant differences between low and high dose inoculated animals (Fig 7E). Correlation of serum cytokine expression with lung viral gRNA did not reveal any significant positive correlation (S3D Fig).

### Survival of K18-hACE2 mice presenting with minimal disease can be modelled by low viral inoculation, which is not affected by NK- or neutrophil depletion

**Fig 8:**
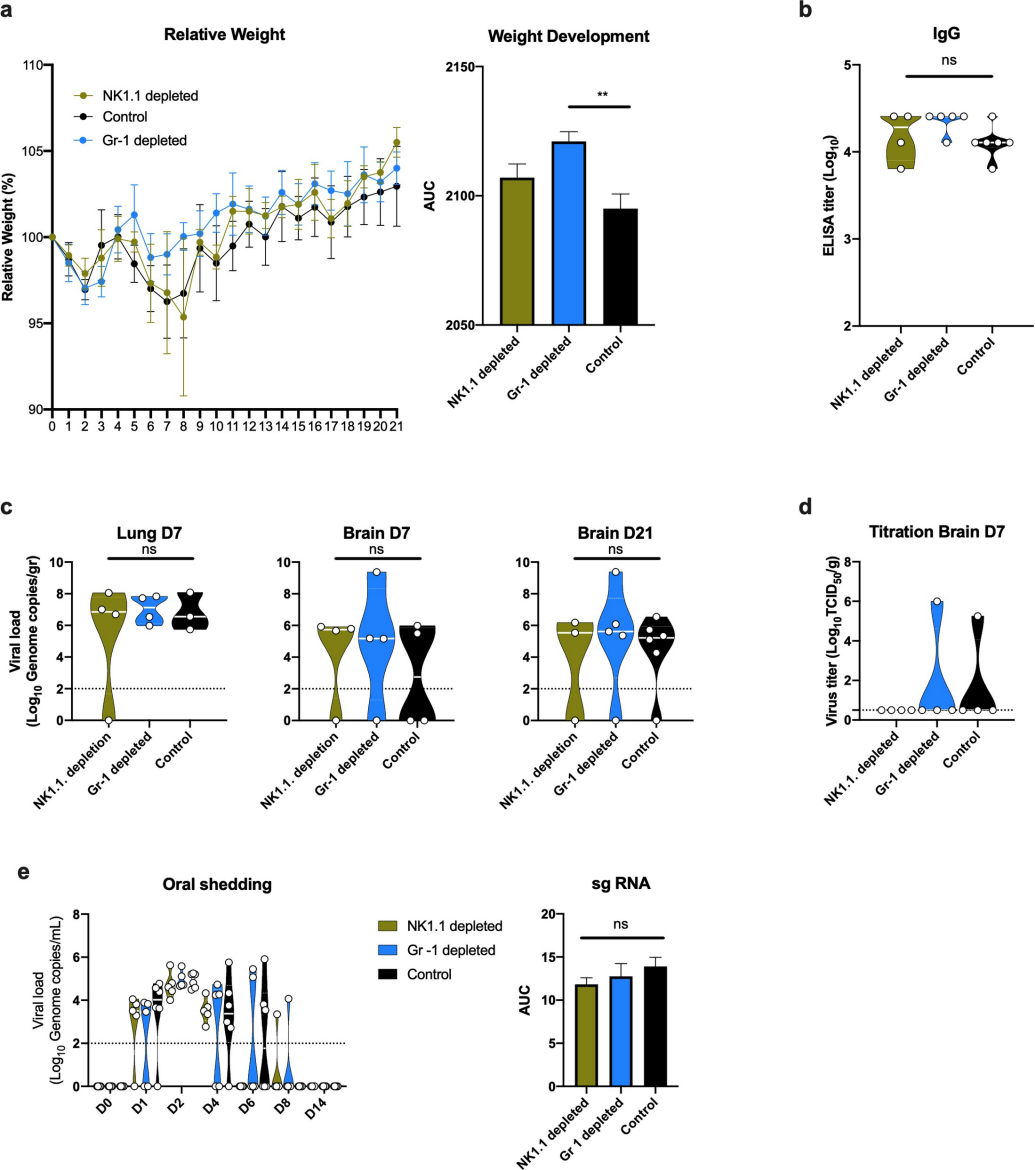
Effect of NK-cell and Gr-1 depletion on SARS-CoV-2 infection in K18-hACE mice. a. Relative weight loss in mice inoculated with 102 TCID50 SARS-CoV-2 after depletion of NK cells or neutrophils (Gr-1+ cells). The lines represent mean ± SEM. Area under the curve (AUC) was determined for each weight curve. b. Violin plot of IgG antibody titres against SARS-CoV-2 spike ectodomain by ELISA in serum. White line represents geometric mean of end point dilutions per study group. Dotted line represents limit of detection. c. Violin plot of viral load in tissues quantified by RT-qPCR with median as centre. d. Violin plot of infectious SARS-CoV-2 titers in brain at 7DPI, with median as centre. Dotted line represents limit of detection. e. Violin plot of viral load in oropharyngeal swabs with median as centre. Viral RNA was quantified using RT-qPCR; Dotted line represents limit of detection. Area under the curve (AUC) was determined for each group. Differences were determined by ordinary one-way ANOVA. Blue: Gr-1 depletion, n = 4); yellow: NK1.1. depletion, n = 4/5); black: control, n = 6).

The observed systemic and respiratory tract chemokine response suggest a strong innate immune response. Increased levels of IFN-γ, IP-10, MCP-1 and TNF-α are associated with severity of disease in COVID-19 patients [25–27]. Neutrophils and NK cells are key mediators of the early innate immune response and have been investigated in the context of COVID-19 severity [28, 29]. To investigate the impact of specific innate immune populations, we selectively depleted male and female K18-hACE mice for NK cells (NK1.1) (13 mice) and neutrophils (Ly6C/Ly6G; Gr-1) (13 mice) or applied the corresponding isotype controls (14 mice). Mice were then intranasally inoculated with 10^2^ TCID_50_ SARS-CoV-2. Depletion efficiency was confirmed on 0 DPI, 3 DPI and 7 DPI (S4 Fig). For each group, 4 mice were sacrificed at 3 DPI and at 7 DPI, the remaining were followed up until 21 DPI. Only one animal succumbed to disease in the NK1.1 depleted group at day 8 DPI, all other mice survived and demonstrated no or minimal signs of disease including slow behavior and unresponsiveness and hunched posture between 7 DPI and 10 DPI. The animal which reached euthanasia criteria presented with a below-normal starting weight, which is likely to have contributed to the more severe disease course. Irrespective of depletion scheme, mice demonstrated a drop in weight at 2 DPI, followed by a second drop in weight from 5 DPI onwards and recovery by 12 DPI (Fig 8A). Comparing the area under the curve for overall weight development post inoculation, Gr-1 depleted animals exhibited significantly less weight loss relative to the control group (Fig 8A) (ordinary one-way ANOVA; p = 0.0037). Seroconversion and presence of spike-specific IgG was observed in all animals remaining at 21 DPI (Fig 8B). On 7 DPI, viral RNA was detected in the lung and brain of most mice, irrespective of group. Interestingly, viral RNA was also detected in the brain of 11 animals at 21 DPI (Fig 8C). Additionally, we found infectious virus in the brain of two animals at 7 DPI and not 21 DPI (Fig 8D). Oropharyngeal swabs were obtained until 14 DPI. SARS-CoV-2 shedding from the respiratory tract was observed in all inoculated animals; mice started shedding at 1 DPI in all three groups and reached peak shedding at 2 DPI (~10^5^ copies/mL), without significant differences between groups (Fig 8E).

## Discussion

In humans, COVID-19 has a broad clinical spectrum ranging from asymptomatic to severe disease [4, 5, 24]. Wildtype mice are not susceptible to infection with SARS-CoV-2 due to an inability of mACE2 to facilitate sufficient cellular entry [16, 17]. Based on existing lethal mouse models for SARS-CoV, first described by McCray and colleagues [19], several transgenic mouse models for COVID-19 have been developed using expression of hACE2 [20, 30–33]. However, mice expressing hACE2 under the mACE2 promoter [20, 30] or exogenously transfected with hACE2 showed only moderate disease with slight weight loss, reduced lung pathology and no lethal phenotype [31, 33]. A mouse model expressing hACE2 under a lung ciliated epithelial cell HFH4 promoter exhibited generally only mild symptoms with lethality observed only in animals with brain infection [32]. In contrast, the K18-hACE2 mouse model described here, which expresses hACE2 under the K18 epithelial promotor, displayed high morbidity and mortality in both high dose and low dose groups. These findings are corroborated by two other studies, currently in preprint [34, 35], which demonstrate a similar disease phenotype in this model.

Previous experiments in different hACE2 mice have demonstrated varying degrees of lung pathology upon infection with SARS-CoV-2 [18, 20–22]. The K18-hACE2 mice developed edema-associated acute lung injury similar to the clinical features of COVID-19 patients, including histological aspects of ARDS. Severe COVID-19 is histologically characterized by diffuse alveolar damage with hyaline membranes, edema, fibrin deposits, multinucleated cells, type II pneumocyte hyperplasia and lymphocyte infiltration composed of a mixture of CD4 and CD8 lymphocytes [36–38]. The analyses of the pathological response observed within the lungs of the SARS-CoV-2 infected mice resemble those observed in humans with regards to lesions and cell tropism.

Even though no neurological or behavioral signs were observed in the animals, SARS-CoV-2 RNA and infectious virus were detected in brain tissues of animals at 7 DPI. SARS-CoV was also reported to infect the brain of K18-ACE2 mice (15,27). This is in line with observations made in HFH4-hACE2 mice and mice expressing hACE2 under control of the murine ACE2 promotor, where viral RNA was also detected in brain tissues [32]. Whether animals died primarily of lung injury and/or brain involvement is not known and could not be answered in this study. However, the presence and absence of virus in the lungs and brain, respectively, by 3 DPI suggest that the virus possibly spreads from respiratory tissues to the brain after I.N. inoculation. Presence of infectious virus in the brain of mice that survived infection at 7 DPI and 21 DPI suggests that the mere presence of virus in the CNS is not indicative of fatal outcome.

In humans, systemic cytokine response to SARS-CoV-2 infection are comprised of TNF-α, IL-1β, IL-1Rα, sIL-2Rα, IL-6, IL-10, IL-17, IL-18, IFN-γ, MCP-3, M-CSF, MIP-1α, G-CSF, IP-10, and MCP-1 [27, 39, 40]. In the lungs of aged hACE2 mice, SARS-CoV-2 infection leads to elevated cytokine production including Eotaxin, G-CSF, IFN-γ, IL-9, and MIP-1β [20]. Here, we show that SARS-CoV-2 infection of K18-hACE2 mice elicits a measurable systemic and local pro-inflammatory cytokine response which is significantly increased at 7 DPI and characterized by an increase in IFN-γ, TNF-α and IP-10, and also encompasses upregulation of innate cell-recruiting chemokines GM-CSF and MCP-1. Importantly, increased levels of IFN-γ, IP-10, MCP-1 and TNF-α are associated with severity of disease in in COVID-19 patients [25–27]. COVID-19 patients also show heightened IL-4 and IL-10 levels, cytokines associated with inhibitory inflammatory responses [41]. While the K18-hACE2 model did not recapitulate IL-4 upregulation, increased IL-10 levels were observed in serum, suggesting that both pro- and anti-inflammatory cytokine response are functioning in this mouse model. This is particularly relevant, as in COVID-19, the resulting cytokine storm is not only thought to be detrimental to disease progression but also closely linked to the development of ARDS [25]. In addition, cytokine levels are also reported to be indicative of extrapulmonary multiple-organ failure [42, 43]. Reports suggest that upregulation of IL-6, IL-8, and TNF-α contributes to SARS-related ARDS [27, 44]. Interestingly, we saw an upregulation of TNF-α in serum, lung and brain, and of IL-6 in lung and brain for high dose animals mostly at 7 DPI, suggesting modulated immune responses and pathogenesis similar to what occurs in humans. The restriction of IL-6 upregulation to the respiratory tracts requires further investigation.

We have also demonstrated a functional humoral immune response and production of both IgM and IgG antibodies by 7 DPI which remained until 21 DPI as observed in the single survivor mouse at 10^4^ TCID_50_ and all survivors at 10^2^ TCID_50_ inoculation. This is in line with observations made in ACE2-HB-01 mice where IgG antibodies against spike protein of SARS-CoV-2 were also observed [30]. This indicates that the K18-hACE2 mouse model mounts a robust innate and adaptive immune response. The early IgG response observed here is in line with observations made in human COVID-19 patients, where simultaneous IgG and IgM seroconversion as early as 2–4 days after symptom onset was described in a subset of patients [45].

It should be acknowledged that the herein presented mouse model relies on the artificial expression of hACE2 under an epithelial promotor, which facilitates an expression pattern of ACE2 that does not precisely recapitulate the human expression pattern [46]. Therefore, the expression of ACE2 in K18-hACE2 mice is not directly influenced by the complex systems regulating endogenous murine ACE2 levels. As such, these mice may not be the most suitable choice for mechanistical studies of pathogenesis. However, this mouse model recapitulates histopathological findings of COVID-19 associated ARDS, neurological involvement, and a robust innate and adaptive immune response conducive to targeted intervention—demonstrated here by the depletion of specific cell populations. Moreover, these mice present a dose-dependent sub-lethal disease manifestation. As such, we believe this model to be highly suitable for testing of SARS-CoV-2 countermeasures such as antiviral and immune-modulatory interventions. However, in humans, COVID-19-associated ARDS presents not only with characteristic lung pathology, but also with clinical manifestations including hypoxia, loss of lung compliance and requirement for intubation, liver and kidney involvement with associated increase in serum protein levels, and decreased lymphocyte numbers. To accurately assess how well K18-hACE2 mice recapitulate human ARDS, additional studies specifically addressing these aspects are required.

## Materials and methods

### Ethics statement

Animal experiment approval was provided by the Institutional Animal Care and Use Committee (IACUC) at Rocky Mountain Laboratories. Animal experiments were executed in an Association for Assessment and Accreditation of Laboratory Animal Care (AALAC)-approved facility by certified staff, following the basic principles and guidelines in the NIH Guide for the Care and Use of Laboratory Animals, the Animal Welfare Act, United States Department of Agriculture and the United States Public Health Service Policy on Humane Care and Use of Laboratory Animals. The Institutional Biosafety Committee (IBC) approved work with infectious SARS-CoV-2 virus strains under BSL3 conditions. All sample inactivation was performed according to IBC approved standard operating procedures for removal of specimens from high containment.

### Cells and virus

SARS-CoV-2 strain nCoV-WA1-2020 (MN985325.1) was provided by CDC, Atlanta, USA. Virus propagation was performed in VeroE6 cells in DMEM supplemented with 2% fetal bovine serum, 1 mM L-glutamine, 50 U/mL penicillin and 50 μg/mL streptomycin. VeroE6 cells were maintained in DMEM supplemented with 10% fetal bovine serum, 1 mM L-glutamine, 50 U/mL penicillin and 50 μg/mL streptomycin.

### Animal experiments

Four to six week-old male and female (15 animals each) transgenic K18-hACE2 mice expressing hACE2 (Jackson laboratories, USA, [19]) were inoculated intranasally (I.N.) with 25 μL sterile Dulbecco's Modified Eagle Medium (DMEM) containing either 10^4^ TCID_50_ (low dose group, n = 14), 10^5^ TCID_50_ (high dose group, n = 14) or 10^5^ TCID_50_ γ-irradiate [47] (control group, n = 2) SARS-CoV-2. At 3 and 7 DPI, four mice from the low dose and high dose groups were euthanized, respectively, and tissues were collected. The remaining mice were utilized for end-point data collection and survival assessment. Mice were weighed and nasal, oropharyngeal and rectal swabs were taken daily. Mice were observed for survival up to 21 DPI or until they reached end-point criteria. End-point criteria included several parameters of severe disease (increased respiratory rate, hunched posture, ruffled fur and lethargy).

For NK-cell and neutrophil depletion experiments, 13 male and female mice, respectively, were first injected with the corresponding depletion antibodies, and 14 mice were given relevant isotype controls. For NK-cell depletion, mice were intraperitoneal administered 1 dose of 150 μg InVivoMAb anti-mouse NK1.1 (PK136) (Bio × Cell) [48] diluted in InVivoPure pH 7.0 Dilution Buffer (Bio × Cell) on -3 DPI and -1 DPI. For neutrophil depletion, mice were administered intraperitoneally 1 dose of 250 μg InVivoMAb anti-mouse Ly6G/Ly6C (Gr-1) (RB6-8C5) (Bio × Cell) [49] diluted in InVivoPure pH 7.0 Dilution Buffer on -1 DPI. Control mice were administered either 2 doses of 150 μg mouse IgG2a isotype control (C1.18.4) or one dose of 250 μg rat IgG2a isotype control (LTF-2), respectively. Mice were bled submandibular to collect baseline samples, then inoculated I.N. with 25 μL sterile DMEM containing 10^2^ TCID_50_ SARS-CoV-2. At 3 and 7 DPI, four mice were euthanized, respectively, and tissues were collected. The remaining mice were followed as described above. Depletion was assessed by flow cytometry at day of inoculation and at 3 and 7 DPI.

### Flow cytometry

Lung tissues were digested with Collagenase type XI (Sigma, 7657) (0.5 mg/mL) and DNAse I type IV (Sigma, D50250) (30 μg/ml) at 37C for 30 min, 500 rpm, then pressed through a 70 μm filter. Blood was collected, erythrocyte lysis was performed for both samples with RBC Lysis Buffer (Biolegend) according to the manufacturer’s description. Viability staining was performed using LIVE/DEADTM Fixable Aqua Dead Cell Stain Kit (Invitrogen). Cells were stained with anti-CD3 (145-2C1), anti-CD45 (30-F11), anti-NK1.1 ((S17016D) and anti-Gr-1 (RB6-8C5) (BD). Samples were fixed overnight in 2.5% paraformaldehyde, data was collected on a BD FACS SymphonyTM A5 and analysed with FlowJoTM Software V10 (BD and Company, 2019). The Gr-1+ population was defined based on viability and on expression of CD45 and Gr-1. The Nk1.1 population was gated as CD45+, CD3- and NK1.1+.

### RNA extraction and quantitative reverse-transcription polymerase chain reaction

Samples were collected with prewetted swabs in 1 mL of DMEM supplemented with 100 U/mL penicillin and 100 μg/mL streptomycin. Then, 140 μL was utilized for RNA extraction using the QIAamp Viral RNA Kit (Qiagen) using QIAcube HT automated system (Qiagen) according to the manufacturer's instructions with an elution volume of 150 μL. Tissues (up to 30 mg) were homogenized in RLT buffer and RNA was extracted using the RNeasy kit (Qiagen) according to the manufacturer's instructions. Viral RNA was detected by qRT-PCR [50]. Five μL RNA was tested with the Rotor-GeneTM probe kit (Qiagen) according to instructions of the manufacturer. Ten-fold dilutions of SARS-CoV-2 standards with known copy numbers were used to construct a standard curve.

### Viral titration

Viable virus in tissue samples was determines as previously described [51]. In brief, tissue samples were weighted, then homogenized in 1 mL of 2% DMEM. SARS-CoV-2 was titrated in quadruplicate in VeroE6 cells; cells were inoculated with ten-fold serial dilutions of tissue homogenate, incubated 1 h at 37°C, the first two dilutions washed twice with 2% DMEM. Cells were incubated with tissue homogenate for 6 days, then scored for cytopathic effect. TCID_50_ was adjusted for tissue weight and calculated by the method of Spearman-Karber.

### SARS-CoV-2 spike glycoprotein enzyme-linked immunosorbent assay (ELISA)

Maxisorp plates (Nunc) were coated with 50 ng spike protein per well and incubated overnight at 4°C. After blocking with casein in phosphate buffered saline (PBS) (ThermoFisher) for 1 h at room temperature (RT), serially diluted 2-fold serum samples (duplicate, in casein) were incubated for 1 h at RT. Spike-specific antibodies were detected with goat anti-mouse IgM or IgG Fc (horseradish peroxidase (HRP)-conjugated, Abcam) for 1 h at RT and visualized with KPL TMB 2-component peroxidase substrate kit (SeraCare, 5120–0047). The reaction was stopped with KPL stop solution (Seracare) and read at 450 nm. Plates were washed 3x with PBS-T (0.1% Tween) in between steps. The threshold for positivity was calculated as the average plus 3x the standard deviation of negative control mouse sera.

### Measurement of cytokines and chemokines

Serum samples, lung and brain homogenate were inactivated with γ-irradiation (2 mRad) and cytokine concentrations were determined on a Bio-Plex 200 instrument (Bio-Rad) using Milliplex Mouse Cytokine/Chemokine MAGNETIC BEAD Premixed 25 Plex Kit (Millipore), according to the manufacturer’s instructions. Samples were pre-diluted 1:3 (serum) and 1:1 (tissues) in the kit serum matrix (v:v). Concentrations below the limit of detections were set to zero; concentrations are depicted as pg/ serum mL or g tissue, respectively. Heatmap and correlation graphs were made in R [52] using pheatmap [53] and corrplot [54] packages.

### Histology and immunohistochemistry

Necropsies and tissue sampling were performed according to IBC-approved protocols. Harvested tissues were fixed for eight days in 10% neutral-buffered formalin, embedded in paraffin, processed using a VIP-6 Tissue Tek (Sakura Finetek, USA) tissue processor, and embedded in Ultraffin paraffin polymer (Cancer Diagnostics, Durham, NC). Samples were sectioned at 5 μm, and resulting slides were stained with hematoxylin and eosin. Specific anti-CoV immunoreactivity was detected using an in-house SARS-CoV-2 nucleocapsid protein rabbit antibody at a 1:1000 dilution. Macrophage (CD68) and T-cell (CD3) immunoreactivities were detected using CD68 rabbit polyclonal antibody (Abcam) at a 1:250 dilution and prediluted CD3 rabbit monoclonal antibody (2GV6, Roche Tissue Diagnostics), respectively. For both CD68 and CD3, ImmPRESS-VR Horse anti-rabbit polymer was used as the secondary antibody (Vector Laboratories). B-cell (CD45) immunoreactivity was detected using anti CD45R rat monoclonal antibody (Abcam) at a 1:500 dilution and ImmPRESS goat anti-rat polymer (Vector Laboratories) as secondary antibody. The immunohistochemistry (IHC) assay was carried out on a Discovery ULTRA automated staining instrument (Roche Tissue Diagnostics) with a Discovery ChromoMap DAB (Ventana Medical Systems) kit. All tissue slides were evaluated by a board-certified veterinary anatomic pathologist blinded to study group allocations.

### Statistical analyses

Two-tailed Mann-Whitney’s rank tests, Wilcoxon matched-pairs rank test, ordinary one-way ANOVA and Kruskal-Wallis test were performed as indicated where appropriate. Statistical significance levels were determined as follows: ns = p > 0.05; * = p ≤ 0.05; ** = p ≤ 0.01; *** = p ≤ 0.001; **** = p ≤ 0.0001.

## Supporting information

S1 Fig Sex-dependent weight loss, mortality and virus shedding in K18-hACE2 mice after SARS-CoV-2 infection. **A.** Body weights were monitored every day. Relative body weight changes are show for female (turquoise) and male (brown) animals for HD (solid) and LD (dotted) groups. **B.** Survival is show for female (turquoise) and male (brown) animals for HD (solid) and LD (dotted) groups. **c**. Nasal, oral and rectal virus shedding in low and high dose infected female (turquoise) and male (brown) mice was quantified by RT-qPCR across time. Individual animals are plotted, violin plot depict median and quantiles. Abbreviations: LD = low dose (10^4^ TCID_50_ SARS-CoV-2), HD = high dose (10^5^ TCID_50_ SARS-CoV-2).
(TIF)

S2 Fig Histologic and immunohistochemical findings in the lungs from a 21 DPI hACE2 transgenic mouse challenged with SARS-CoV-2. **A**. Multiple foci of perivascular inflammation and increased alveolar cellularity. **B**. Perivascular and peribronchiolar lymphocytic inflammation. **C**. Aggregated lymphocytes within alveolar septa and alveoli containing foamy macrophages. **D**. Foamy macrophages cluster and fill alveoli and alveolar septa contain increased numbers of lymphocytes. **E**. CD68 immunoreactivity in foamy alveolar macrophages. **F**. One of many discreet aggregates of lymphocytes in the 21 DPI lung composed of **G**. CD45+ B cells and **H**. CD3+ T cells. Magnification: a = 40x; b, c, f, g, h = 200x; d, e = 400x.
(TIF)

S3 Fig Multiplex analysis of cytokines/chemokines in K18-hACE mice challenged with SARS-CoV-2 measured at 3- and 7-days post inoculation. **A**. Individual animals are plotted, violin plots depict median and quantiles. Low dose = blue, high dose = red. **B**. Correlation between cytokine levels and viral RNA in the lungs. Significant correlations (p = 0.05) are shown and strength of correlation is depicted according to the colour bar, crossed bars are not significant. Abbreviations: DPI = days post inoculation, G-CSF = granulocyte colony-stimulating factor, GM-CSF = granulocyte-macrophage colony-stimulating factor, INF = interferon, IL = interleukin, KC = keratinocyte chemoattractant, MCP = monocyte chemoattractant protein, MIP = macrophage inflammatory protein, IP = interferon-γ-inducible protein, TNF = tumour necrosis factor.
(TIF)

S4 Fig Depletion efficiency of NK-cell and Gr-1 depletion in SARS-CoV-2 infection in K18-hACE mice. A. Depletion efficiency was determined on 0, 3 and 7 DPI in blood and on 3 and 7 DPI in lung. Gr-1 population (bottom) was defined as CD45+ Gr-1+, NK1.1 population (top) as CD45+, CD3- and NK1.1+. Differences to control animals were determined by Kruskal-Wallis test.
(TIF)

S1 Table Histologic findings in K18 hACE2 mice associated with SARS-CoV-2 infection. (XLSX)
