# Supplementary figures and images for: K18-hACE2 mice develop respiratory disease resembling severe COVID-19
Source: PLoS Pathog. 2021 Jan 19;17(1):e1009195. doi: 10.1371/journal.ppat.1009195 (PMC7875348; doi:10.1371/journal.ppat.1009195)

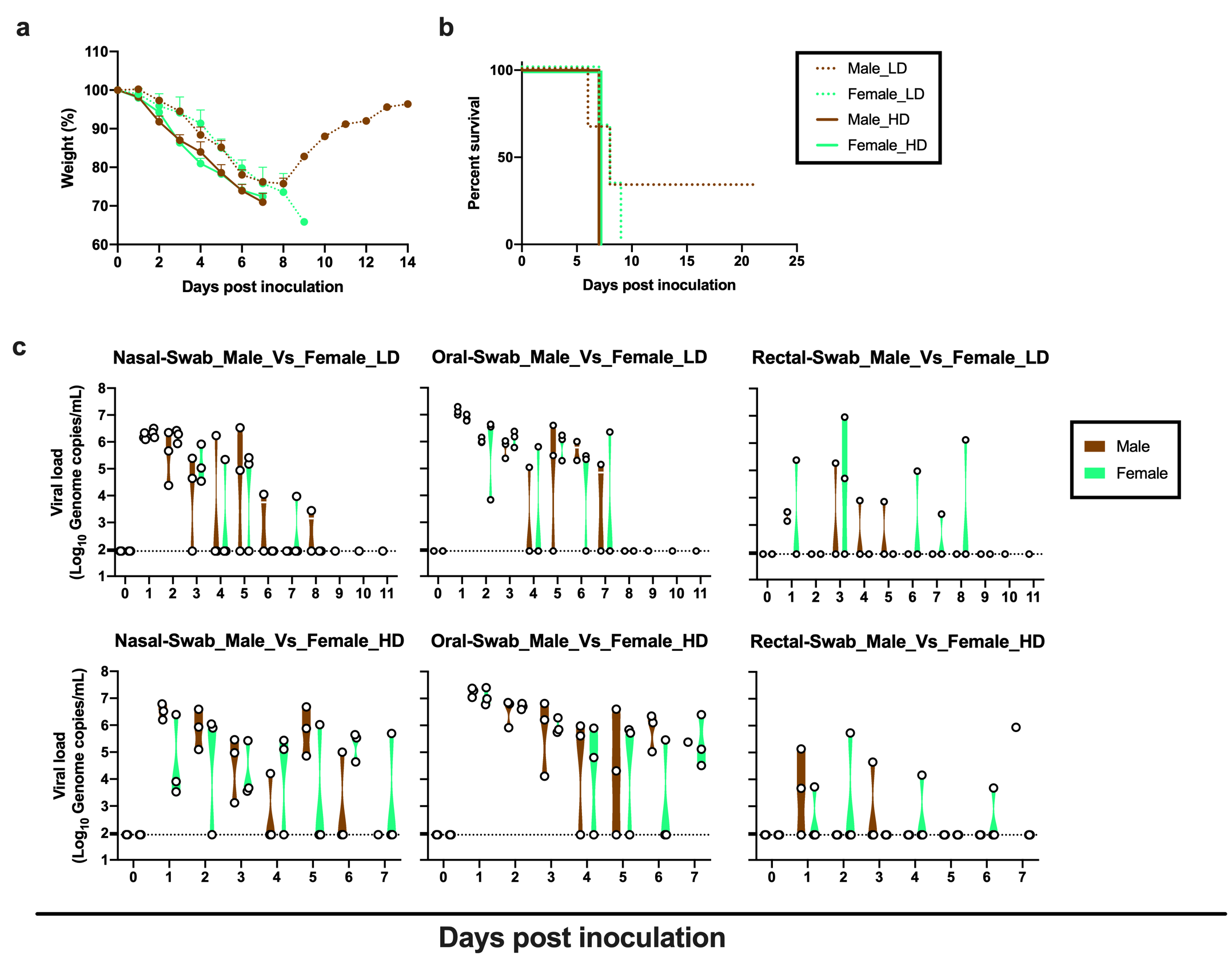

Supplement: S1 Fig — A. Body weights were monitored every day. Relative body weight changes are show for female (turquoise) and male (brown) animals for HD (solid) and LD (dotted) groups. B. Survival is show for female (turquoise) and male (brown) animals for HD (solid) and LD (dotted) groups. c. Nasal, oral and rectal virus shedding in low and high dose infected female (turquoise) and male (brown) mice was quantified by RT-qPCR across time. Individual animals are plotted, violin plot depict median and quantiles. Abbreviations: LD = low dose (104 TCID50 SARS-CoV-2), HD = high dose (105 TCID50 SARS-CoV-2). (TIF) [file ppat.1009195.s001.tif]

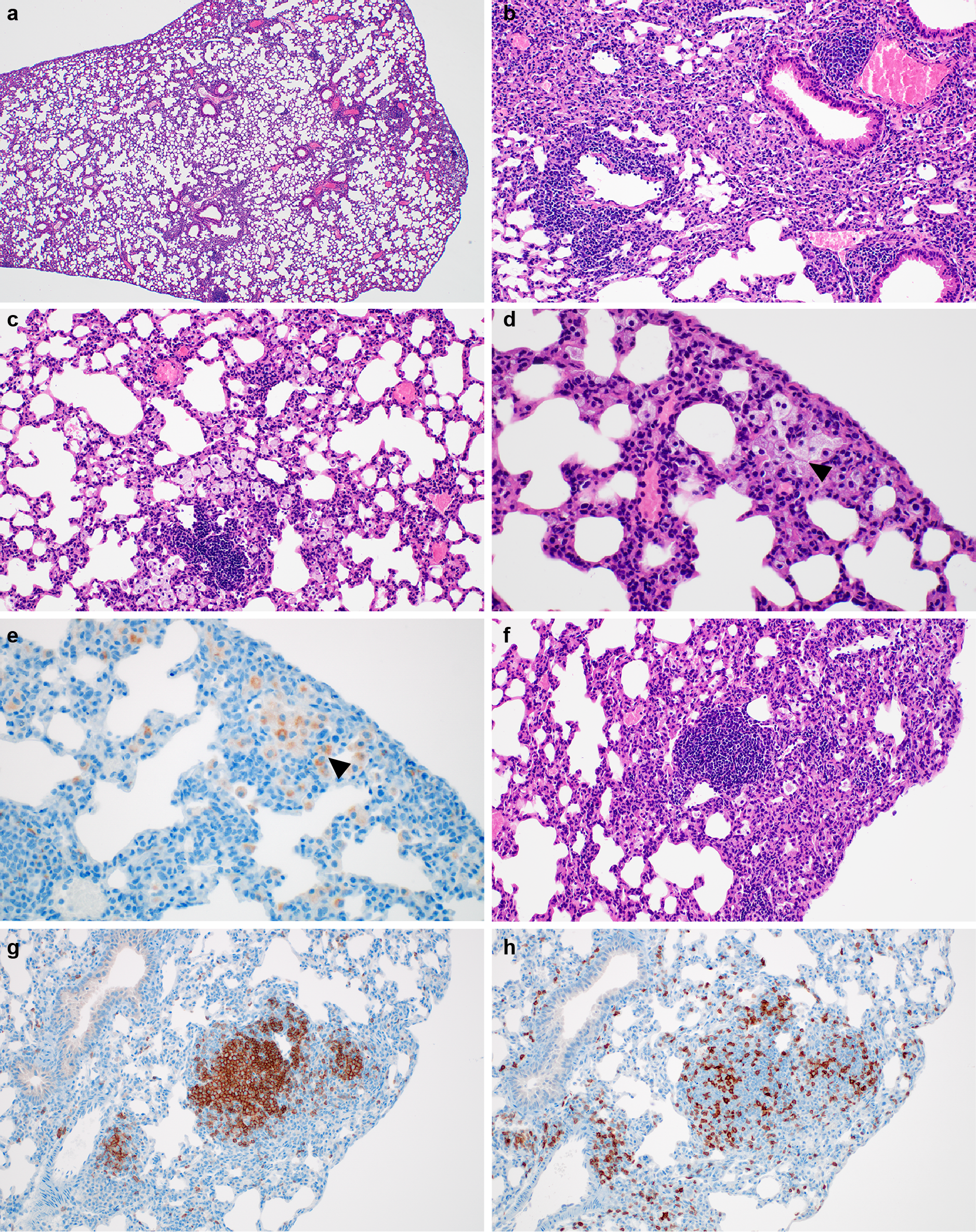

Supplement: S2 Fig — A. Multiple foci of perivascular inflammation and increased alveolar cellularity. B. Perivascular and peribronchiolar lymphocytic inflammation. C. Aggregated lymphocytes within alveolar septa and alveoli containing foamy macrophages. D. Foamy macrophages cluster and fill alveoli and alveolar septa contain increased numbers of lymphocytes. E. CD68 immunoreactivity in foamy alveolar macrophages. F. One of many discreet aggregates of lymphocytes in the 21 DPI lung composed of G. CD45+ B cells and H. CD3+ T cells. Magnification: a = 40x; b, c, f, g, h = 200x; d, e = 400x. (TIF) [file ppat.1009195.s002.tif]

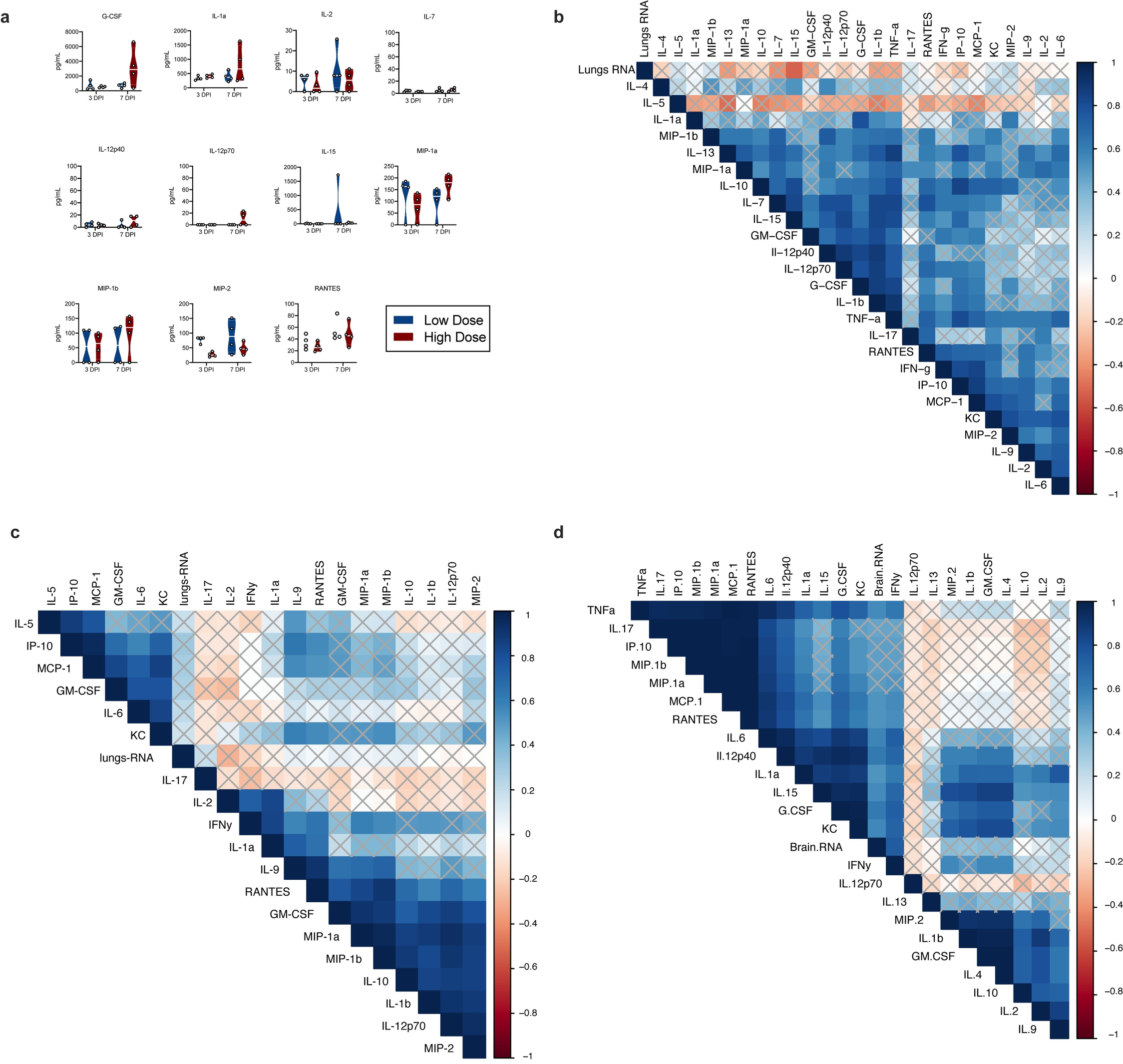

Supplement: S3 Fig — A. Individual animals are plotted, violin plots depict median and quantiles. Low dose = blue, high dose = red. B. Correlation between cytokine levels and viral RNA in the lungs. Significant correlations (p = 0.05) are shown and strength of correlation is depicted according to the colour bar, crossed bars are not significant. Abbreviations: DPI = days post inoculation, G-CSF = granulocyte colony-stimulating factor, GM-CSF = granulocyte-macrophage colony-stimulating factor, INF = interferon, IL = interleukin, KC = keratinocyte chemoattractant, MCP = monocyte chemoattractant protein, MIP = macrophage inflammatory protein, IP = interferon-γ-inducible protein, TNF = tumour necrosis factor. (TIF) [file ppat.1009195.s003.tif]

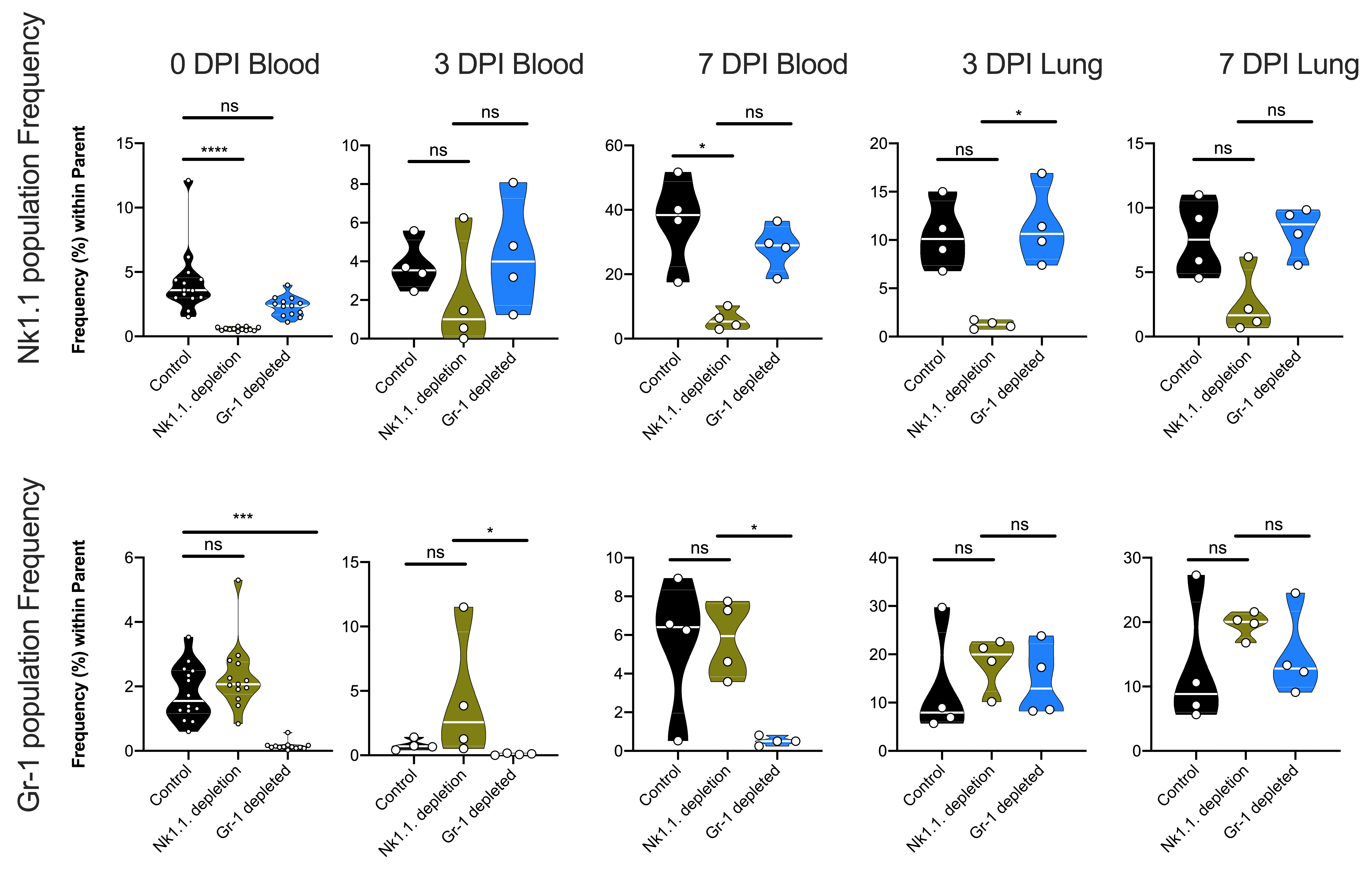

Supplement: S4 Fig — A. Depletion efficiency was determined on 0, 3 and 7 DPI in blood and on 3 and 7 DPI in lung. Gr-1 population (bottom) was defined as CD45+ Gr-1+, NK1.1 population (top) as CD45+, CD3- and NK1.1+. Differences to control animals were determined by Kruskal-Wallis test. (TIF) [file ppat.1009195.s004.tif]
